# Classification and challenges in the histopathological diagnosis of peripheral T-cell lymphomas, emphasis on the WHO-HAEM5 updates

**DOI:** 10.3389/fonc.2022.1099265

**Published:** 2022-12-20

**Authors:** Carlos Murga-Zamalloa, Kedar Inamdar

**Affiliations:** ^1^ Department of Pathology, University of Illinois at Chicago, Chicago, IL, United States; ^2^ Department of Pathology, Henry Ford Hospital, Detroit, MI, United States

**Keywords:** T-cell lymphoma, WHO, classification, diagnosis, molecular diagnosis, biology, pathology

## Abstract

Mature T-cell lymphomas represent neoplastic expansions of T-cell lymphocytes with a post-thymic derivation. Most of these tumors feature aggressive clinical behavior and challenging histopathological diagnosis and classification. Novel findings in the genomic landscape of T-cell lymphomas are helping to improve the understanding of the biology and the molecular mechanisms that underly its clinical behavior. The most recent WHO-HAEM5 classification of hematolymphoid tumors introduced novel molecular and histopathological findings that will aid in the diagnostic classification of this group of neoplasms. The current review article summarizes the most relevant diagnostic features of peripheral T-cell lymphomas with an emphasis on the updates that are incorporated at the WHO-HAEM5.

## 1 Introduction

**Figure 1:**
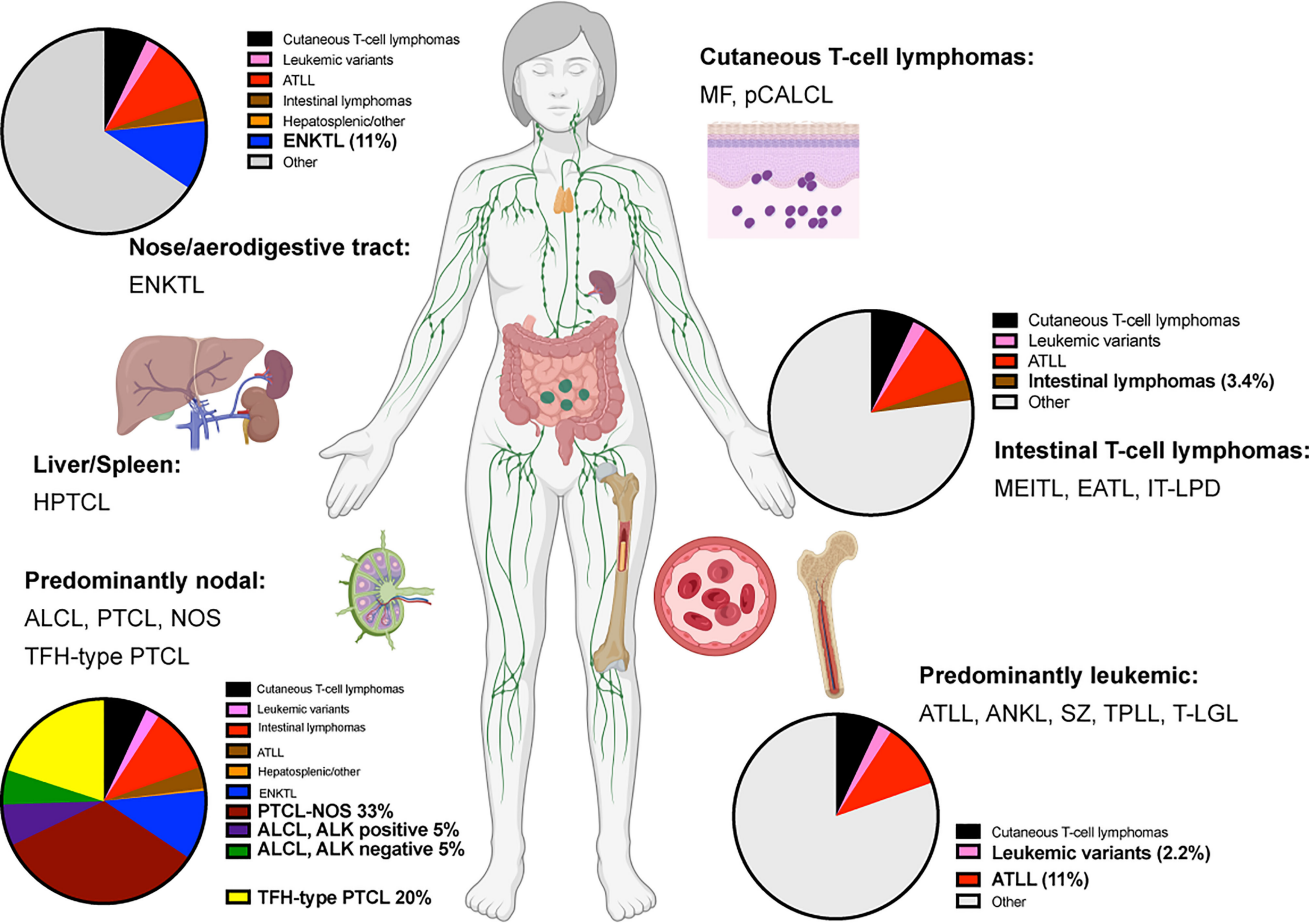
Predominant anatomical presentation and relative frequency of mature T-cell lymphomas. Abbreviations: Extranodal T/NK-cell lymphoma, nasal type (ENKTL); Hepatosplenic T-cell lymphoma (HPTCL); Anaplastic large cell lymphoma (ALCL); Peripheral T-cell lymphoma, non-otherwise specified (PTCL, NOS); T-follicular helper type peripheral T-cell lymphoma (TFH-PTCL); Mycosis Fungoides (MF); Primary cutaneous anaplastic large cell lymphoma (pCALCL); Monomorphic epitheliotropic intestinal T-cell lymphoma (MEITL), Enteropathy-associated T-cell lymphoma (EATL); Indolent T-cell lymphoproliferative disease of the gastrointestinal tract (IT-LPD); Adult T-cell leukemia/lymphoma (ATLL); Aggressive NK-cell leukemia (ANKL); Sèzary Syndrome (SZ); T-cell prolymphocytic lymphoma (T-PLL); T-cell large granular lymphoma (T-LGL).

Mature T and NK cell lymphomas represent approximately 5-10% of all non-Hodgkin lymphomas globally. With few exceptions, these groups of neoplasms are usually diagnosed at an advanced stage in adult patients. They feature an aggressive clinical course, with overall survival of 3 to 5 years after initial diagnosis (1–6). Currently, more than 20 different types of mature T-cell lymphomas (excluding primary cutaneous T-cell lymphomas) exist (**Figure 1**). The diagnosis is usually challenging due to the lack of specific molecular markers and overlapping morphological features.

The continuous discovery of the genomic landscape and mutational signatures of T-cell lymphomas is helping to identify novel molecular biomarkers that will improve the classification, patient risk stratification and introduce novel tailored therapies. The current review focuses on the relevant pathological and molecular findings that were incorporated in the upcoming World Health Organization classification of Haematolymphoid Tumours (WHO-HAEM5) (7). The updated WHO-HAEM5 has introduced organizational changes that aid in the differential diagnosis of mature T-cell lymphomas with a leukemic presentation. Modifications in the terminology of entities such as indolent T-cell lymphoproliferative disease of the GI tract and peripheral T-cell lymphoma of T-follicular helper origin are also included. Finally, the WHO-HAEM5 included novel genomic findings to help recognize aggressive forms in entities such as T-cell large granular leukemia (T-LGL) and Adult T-cell leukemia/lymphoma (ATLL).

## 2 Mature T-cell and NK-cell leukemias

This section encompasses the mature T-cell neoplasms characterized by predominant leukemic presentation (**Table 1**). This section also included Sèzary Syndrome to emphasize the primary site of presentation.

**Table 1:** List of disease entities with a predominant leukemic presentation.

Mature T-cell and NK-cell leukemias				
T-cell prolymphocytic leukemia (T-PLL)				
T-cell large granular lymphocytic leukemia				
NK-large granular lymphocytic leukemia				
Adult T-cell leukemia/lymphoma				
Sezary Syndrome				
Aggressive NK-cell leukemia

### 2.1 T-cell prolymphocytic leukemia

Is characterized by the leukemic expansion of neoplastic post-thymic T-cell lymphocytes. More than 90% of the cases feature chromosomal abnormalities that involve the 14q32 or Xq28 loci associated with the increased cytoplasmic expression of TCL1A, TCL1B, or MTCP1. Immunophenotypically, the tumor cells are characterized by CD4 expression in 75% of the cases and positive expression of both CD4 and CD8 markers in 25% of the cases (8, 9). The WHO-HAEM5 incorporated diagnostic recommendations from the T-PLL international study group (TPLL-ISG) assembled in 2017 (10). The proposed guidelines indicate that at least 2 (out of 3) major criteria must be met to establish a diagnosis of T-PLL (**Table 2**). The major criteria include defining T-PLL characteristics, including peripheral blood/bone marrow involvement, specific genetic aberrancies (*TCL1A/B* and *MTCP*), and T-cell clonality (**Table 2**). Importantly, less than 10% of the cases will feature characteristic clinical features of T-PLL, yet genetic aberrancies of *TCL1A*, *TCL1B*, or *MTCP* are not identified. This group of patients is recognized as TCL1-family negative T-PLL. In these scenarios, the proposed diagnostic criteria indicate that at least one chromosomal abnormality specific to T-PLL or the involvement of T-PLL-specific sites (spleen, effusions, skin, and CNS) must be present to establish a definitive diagnosis of T-PLL (1 minor criteria, **Table 2**).

**Table 2:** Proposed diagnostic criteria for T-PLL by the T-PLL international study group (TPLL-ISG). *Adapted from Staber et al.* (10).

Diagnostic criteria for T-PLL
Major criteria
≥ 5 x 10^9^/L cells of with a T-PLL phenotype in the peripheral blood or bone marrow
Evidence of T-cell clonality
Abnormalities of 14q32 or Xq28 or expression of TCL1A, TCL1B or MTCP1
Minor criteria
Abnormalities involving the chromosome 11 (11q22.3; ATM)
Abnormalities in chromosome 8: idic (8)(p11), t(8:8), trisomy 8q
Abnormalities in chromosome 5, del12p, 13, 22 or a complex karyotype
Involvement of a T-PLL specific site (splenomegaly, effusions, skin and CNS)

### 2.2 T-cell large granular lymphocytic leukemia

Is defined by a persistent (at least 6 months) increase in the number of circulating large granular lymphocytes (2-20 x 10^9^/L). About 40-50% of the patients will develop neutropenia, 30-40% anemias, and 10-25% splenomegaly. The updated WHO-HAEM5 incorporated novel molecular findings from two studies that established a correlation between the genomic landscape and clinical characteristics.

The first study (Sannikommu et al.) conducted a retrospective analysis of 224 patients with T-LGL (median follow-up of 36 months). It evaluated the clinical outcomes, therapy responses, and the mutational status of *STAT3 (* 11). In this study, mutations in *STAT3* were identified in 36% of the patients, and the most frequent change was Y640F-STAT3. The study demonstrated that patients with *STAT3* mutations are more likely to feature neutropenia and anemias. However, no differences in overall survival were identified when patients were stratified according to the mutational status of *STAT3*.

The second (Barila et al.) is a retrospective study that evaluated 169 patients diagnosed with T-LGL between 1992 and 2018 (12). *STAT3* mutations were identified in 60% of the patients with CD8+ T-LGL (n=65). However, mutations in *STAT3* were not detected in patients with CD4+/CD8^neg/dim^ T-LGL. *STAT5b* mutations were detected in 34% of CD4+/CD8^neg/dim^ T-LGL patients and not in CD8+ T-LGL patients. Correlation with clinical characteristics showed that patients with *STAT3* mutations were characterized by a higher frequency of neutropenia, anemia, and transfusion-dependent anemia. Multivariate analysis demonstrated that mutations in *STAT3* were independently associated with reduced overall survival (267 months vs. not-reached).

### 2.3 Chronic lymphoproliferative disorder of natural killer cells 

The study by Barila et al. also included 36 patients with CLPD-NK (12). The clinical characteristics of CLPD-NK were like the T-LGL counterparts. Approximately 26% of the patients featured anemia, 48% neutropenia, and 13% splenomegaly. However, mutations in *STAT3* were identified only in 6% of the patients, and *STAT5b* mutations were not detected. Expression of CD94 was identified in 94% of the CLPD-NK cases. In contrast, CD94 expression was detected in 14% of Tα/β T-LGL and 52% of Tγ/δ T-LGL cases. Due to the clinical similarities with T-LGL, the WHO-HAEM5 has renamed this entity NK-large granular lymphocytic leukemia (NK-LGL).

### 2.4 Adult T-cell leukemia/lymphoma

Is an aggressive T-cell neoplasm caused by infection of the human T-cell leukemia virus type 1 (HTLV-1). Therefore, it is predominantly diagnosed in HTLV-1 endemic areas including Japan, Central America, South America, and intertropical Africa (13). The neoplastic lymphocytes show variable morphology and range from small to large forms, with irregular nuclear contours, ‘flower-like cells,’ and occasionally vacuolated cytoplasm (14). CD3/CD4 positive, mature T-cells characterize the immunophenotype with co-expression of CD25, FoxP3, and lack of CD7 expression (14). The most common presentation (acute type, ~60%) and the chronic variant (~15% of cases) are characterized by leukemic involvement with hepatosplenomegaly. Predominantly nodal and cutaneous manifestations occur in the smoldering and lymphomatous variants (~25% of cases). Those two variants are more frequent in geographical areas where HTLV-1 is nonendemic, and the diagnosis is usually problematic because those subtypes can mimic cutaneous T-cell lymphomas or, more rarely, anaplastic large cell lymphoma (15). The prognosis is poor, regardless of the clinical presentation, and ranges from 1 year to 2 years after diagnosis. HTLV-1 serology testing is non-diagnostic of ATLL, and direct detection of viral transcripts in the neoplastic cells is critical for a definitive diagnosis in cases where the clinical and histopathological presentation is suggestive of different subtypes of T-cell lymphomas (15, 16).

The updated version of WHO-HAEM5 has incorporated novel genomic findings from two recent studies. The first study (Kogure et al.) identified recurrent loss-of-function mutations in the CIC-L/ATXN1 complex after performing whole-exome sequencing in 150 ATLL patients (17). The *CIC-L* gene encodes a transcriptional repressor that complexes with the ATXN1 protein. Mutations or structural variations in *CIC-L* and *ATXN1* were mutually exclusive in ATLL patients, and in combination, alterations in the ATXN1 and CIC-L complex were identified in 53% of the cases (17). To support the oncogenic role of CIC-L/ATXN1 during the neoplastic expansion of ATLL, murine models demonstrated that conditional deletion of *CIC-L* in CD4+ T-cell lymphocytes was associated with the proliferation of CD4+CD25+CD127-FoxP3+ regulatory T-cells.

A second study evaluated the clinical outcomes and the genetic landscape of 463 ATLL patients (18). Patients with aggressive forms of ATLL displayed a higher number of mutations. The 3-year overall survival rate was 54% for patients with 0 to 1 mutation, 39% for those with 2-5 mutations, and 19% for those with more than 6 mutations (18). Multivariate analysis demonstrated that older age (more than 70 years), *PRKCB* mutations, and *PD-L1* amplifications were independent prognostic factors for poor overall survival. Copy number amplifications of *PD-L1* were more frequent in the aggressive group and were predictive of poor outcomes in patients with indolent and aggressive forms of the disease. Among the patients with indolent clinical behavior, *IRF4* mutations, *PD-L1* amplifications, and *CDKN2A* deletions predicted poor outcomes (18). Overall, these findings highlight the relevance of including genomic profiling for the classification and prognosis of ATLL.

### 2.5 Aggressive NK-cell leukemia

Is a rare NK-cell neoplasm characterized by a fulminant clinical course with a median overall survival of 2 months or less after diagnosis. A leukemic presentation and occasional skin and CNS involvement characterize this entity. The WHO-HAEM5 included under this category the cases of NK/T cell lymphoma with an intravascular presentation that were previously considered a variant of extranodal NK/T-cell type lymphoma. In addition, novel clinical findings and mutational analysis are included under this category in the updated WHO-HAEM5.

A recent large retrospective evaluated the molecular profile and clinical features of 161 individuals diagnosed with ANKL in China (19). The peak incidence was 21 and 30 years old, with a median overall survival of 55 days. A group of the patients (16%) demonstrated a prolonged prodromal disease (subacute ANKL) that was characterized by fever, lymphocytosis, generalized lymphadenopathy, and hepatosplenomegaly (infectious mononucleosis-like symptoms). The prodromal phase has a median duration of 115 days, precedes the fulminant onset of ANKL, and the overall mean survival in this group of patients is 213 days. In contrast, patients without a prodromal phase (‘classic’ ANKL) have a median overall survival of 44 days. A targeted sequencing panel identified similar frequencies of mutations in the JAK-STAT pathway between the two groups. Importantly, mutations in *TP53* were detected in 38% of the ‘classic’ ANKL patients, and no mutations in *TP53* were identified in the subacute group (19).

A second study evaluated the genomic landscape of 10 ANKL patients utilizing whole-exome sequencing. The mutational spectrum of ANKL clustered separately from other related mature T-cell lymphomas with leukemic presentation (T-LGLL, T-PLL, and CPLD-NK) (20). The most recurrently mutated genes were *DDX3X* (29%) and *STAT3* (21%), and mutations in TP53 were detected in a single patient. The most frequent mutations in ANKL were also seen in NK-T-cell lymphoma, nasal type (NKTCL); therefore, no specific mutations were identified in the ANKL that can help distinguish those cases from NKTCL (20).

## 3 Intestinal T-cell lymphomas

**Figure 2:**
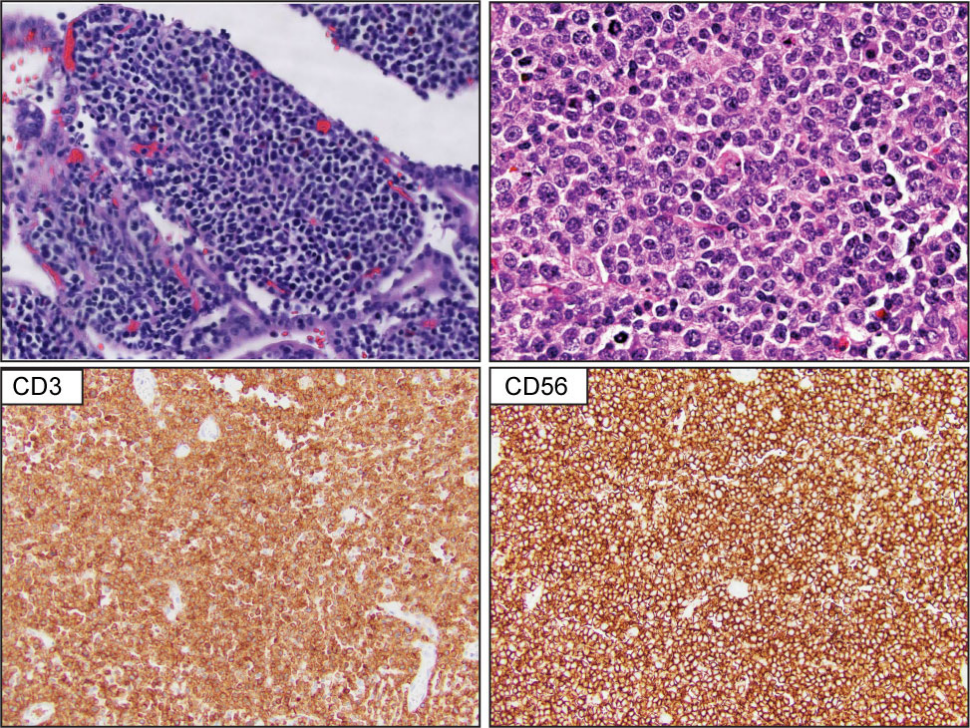
Monomorphic epitheliotropic intestinal T-cell lymphoma (MEITL). Upper panel show representative H&E images that highlight extensive epitheliotropism by medium size lymphocytes with a homogeneous appearance. The lower panel demonstrates that the atypical lymphocytes are composed of CD3-positive and CD56-positive T-cell lymphocytes.

**Figure 3:**
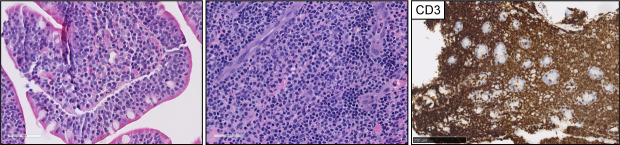
Intestinal T-cell lymphoma, non-otherwise specified (ITCL-NOS). H&E pictures demonstrate dense infiltrates of medium to large, atypical lymphocytes with epitheliotropism. The infiltrates were composed of CD3-positive T-cell lymphocytes with (not shown) aberrant CD7 and CD5 loss and negative expression of CD56.

Primary intestinal T-cell lymphomas constitute a diverse group of neoplasms predominantly derived from resident intestinal T-cell lymphocytes. Apart from NK-cell enteropathy, these neoplasms feature an aggressive clinical course and a poor prognosis. Within this group, the neoplastic proliferations that arise secondary to celiac disease are defined as enteropathy-associated T-cell lymphoma (EATL). EATL is an aggressive disease predominantly composed of CD8+ T-cells with the expression of TCR-α/β (21, 22). Monomorphic epitheliotropic intestinal T-cell lymphoma (MEITL) is more frequent in Asia and is composed of medium-sized lymphocytes with a homogeneous appearance, epitheliotropism, and frequent expression of CD56 (23–27) (**Figure 2**). The category of intestinal T-cell lymphomas non-otherwise specified (ITCL-NOS) remains as an entity that fails classification with the current schemes (**Table 3**; **Figure 3**), with very few case reports available (28–30). The WHO-HAEM5 has modified the designation of indolent-type T-cell lymphoproliferative disease of the GI tract and has incorporated novel molecular findings in NK-cell enteropathy.

**Table 3:** Histological and molecular features of intestinal T-cell lymphomas. *Adapted from Osmani et al.* (27).

	NK-cell lymphoproliferative disorder of the GI tract	Indolent T cell lymphoma of the GI tract	EATL	MEITL
**Epidemiology**	*Unknown*	*Unknown*	Northern Europe	Asian, Hispanic
**Associations**	*Unknown*	*Unknown*	Celiac, HLA-DQ2, HLA-DQ8	*Unknown*
**Location**	Stomach, small intestine, colon	Small intestine, colon, others	Small intestine	Small intestine
**Histology**	Medium to large in size, with mild cytological atypia. Destruction of adjacent glands may be present at advanced stages. Epitheliotropism is usually absent.	Small and monotonous lymphocytes, with none to mild cytological atypia. Non-destructive. Occasional epitheliotropism.	Pleomorphic. Medium to large with cytological atypia. Epitheliotropism is usually present. Angiodestruction may be present.	Monomorphic medium cells. Epitheliotropism usually present.
**Phenotype**	cCD3+* CD8- CD5- CD7+ CD4- CD56+ TIA1+ EBER(ish)- Granzyme B+ Low Ki-67(<25%). Increase levels of pSTAT5.	CD2+ CD3+ CD5+/- CD7+/- CD8+>CD4+*** CD56- TIA1-/+ Granzyme B- EBER(ish) - Low Ki-67 (<10%)	CD3+ CD4- CD5- CD7+ CD8-/+**** CD56- CD103+ CD30+/- TIA-1+, Granzyme B+ High Ki-67 (>50%)	CD3+ CD5- CD8+>CD4+ CD56+** CD30- CD103+ TIA1+ Granzyme B+ EBER(ish)- High Ki-67 (>50%). MATK+^#^
**TCR expression**	Negative	Alpha beta (αβ)	Alpha beta (αβ) > gamma delta (ɣδ)	Gamma delta (ɣδ) > Alpha beta (αβ)
**Molecular**	TCR polyclonal. JAK3 mutations.	STAT3-JAK2 fusion	STAT5B, JAK3, GNAI2 Gains of 1q and 5q	SETD2, STAT5B, JAK3, GNAI2 Gains of MYC

Differential diagnosis to Intestinal T cell lymphomas. *Concurrent flow cytometry analysis demonstrates that CD3 expression is cytoplasmic. **Majority of tumors (~80%) are CD8+, a small subset of cases (9-18%) can be negative for CD56. *** Majority of the cases are CD8+ (~80%), a minority of the cases are either CD4+ or double CD4/CD8 negative in similar proportions. **** Approximately 30% of the cases are CD8+, majority of the cases are CD4-/CD8-. ^#^MATK/Lsk nuclear expression is detected in majority of MEITL cases and in NK/T cell lymphomas, whereas EATL cases only feature cytoplasmic staining.

### 3.1 Indolent T-cell lymphoproliferative disease of the gastrointestinal tract

Is morphologically characterized by superficial infiltrates composed of small lymphocytes with minimal cytological atypia that exhibit a Ki-67 proliferation index that is less than 10% (31). This entity is characterized by chronic and relapsing gastrointestinal symptoms, including diarrhea, dyspepsia, and vomiting (32, 33). In some cases, disease progression with an aggressive clinical course has been reported (34, 35). Therefore, the term ‘lymphoproliferative’ has been replaced by ‘lymphoma’ to highlight that this entity represents a clonal disease with a risk of transformation into more aggressive forms.

### 3.2 NK-cell enteropathy

Has been renamed indolent NK-cell lymphoproliferative disorder of the GI tract (iNKLPD). This entity is characterized by non-specific gastrointestinal symptoms, including abdominal pain, constipation, and reflux, with a chronic/relapsing course (36). The tumor cells feature mild cytological atypia, and the Ki-67 proliferation index is usually in the 25%-50% range; in contrast to ENKTCL, these tumors are negative for EBV expression. Additional case series reports have confirmed the indolent clinical course, with no reported cases of progression to more advanced stages (37). A recent study in 7 patients identified somatic mutations in *JAK3* in 30% of the patients; additional mutations in other genes, including *IFG1R* and *AURKB*, were also detected at lower frequencies (38). The same study demonstrated increased expression of phosphorylated STAT5 in 100% of cases (n = 7) (38), supporting the idea that this process constitutes a neoplastic lymphoproliferative disease.

## 4 Nodal T-cell lymphomas

Nodal involvement is the most frequent mode of presentation of T-cell lymphomas, and the three entities discussed below account for more than 60% of all mature T-cell neoplasms. Secondary nodal involvement by other T-cell lymphomas, such as ENKTL (discussed in 5.1) or cutaneous T-cell lymphomas, is not uncommon. However, their primary presentation site is different from nodal, and the involvement of lymph nodes can occur during the evolution of the disease.

### 4.1 Anaplastic large cell lymphoma

**Figure 4:**
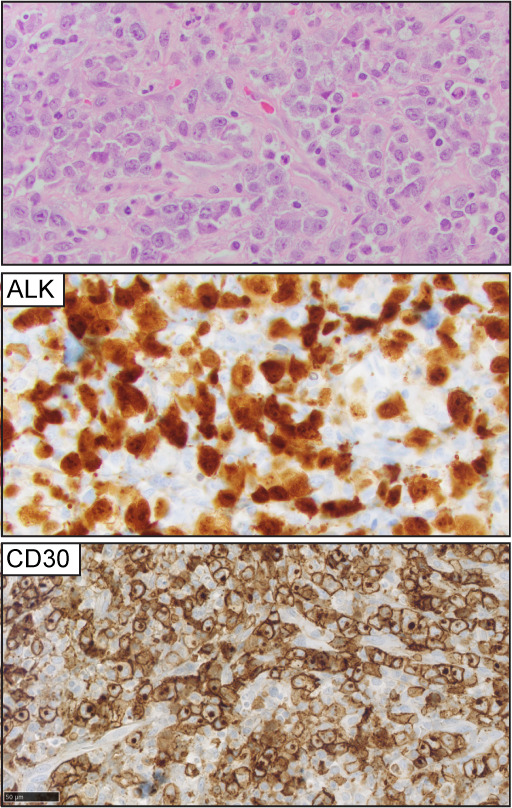
Anaplastic large cell lymphoma (ALCL). Representative (H&E) pictures of ALCL highlight the clustering of large, atypical lymphocytes with a variable amount of cytoplasm, open chromatin, and ‘kidney-shaped’ nuclei. ALK expression is detected in 50% of the cases (ALK+ ALCL). CD30 expression is homogeneously positive in at least 80% of the tumor cells.

ALCL is the third most common nodal T-cell lymphoma and accounts for approximately 11-13% of peripheral T-cell lymphomas (39, 40). Morphologically is characterized by large anaplastic cells, ‘hallmark cells,’ organized in a cohesive pattern, with uniform and strong expression of CD30 in more than 80% of the tumor cells (41) (**Figure 4**). Translocations involving the tyrosine kinase ALK are present in approximately 50% of ALCL cases. The uncontrolled activation of the ALK-kinase defines a phosphoproteomic and transcriptional signature that drives the oncogenic program of ALK+ ALCL cases (42–44), and ALK+ ALCL constitutes a specified entity. ALK-negative ALCL comprises a heterogenous group and is characterized by inferior clinical outcomes compared to ALK+ ALCL. Specific genomic rearrangements within this group, including *DUSP22*, and *TP63* fusions, are associated with differential clinical outcomes (45–48). The WHO-HAEM5 has included novel genomic findings from two studies that will help understand the biology of ALCL.

Hapgood et al. (48) correlated the outcomes and molecular features of 62 ALK-negative ALCL patients. The findings demonstrated inferior clinical outcomes in patients with *DUSP22* rearrangements (n = 12, 44% five-year OS) in comparison to ≥80% 5-year overall survival (OS) that was described in the previous case series (47). These findings indicate that additional molecular mechanisms can modify the outcomes of these patients. Consistent with this, a recent cohort study in 82 ALCL patients with systemic ALCL (ALK+ and ALK-) identified that mutations in *TP53* and *STAT3* are associated with a worse prognosis independent of ALK status (49).

The second study demonstrated the presence of specific super-enhancer (SE) regions enriched with *BATF3* sites in ALCL cell lines and patient samples. The findings further revealed that the transcriptional activity of *BATF3* can mediate the expression of IL2R. Importantly, increased expression of IL2R was predominantly detected in ALCL cases compared to different peripheral T-cell lymphomas, including AITL and PTCL-NOS. The findings indicate that chromatin remodeling may play a critical role during the growth of these groups of tumors and that IL2-dependent signaling plays a pivotal role in substituting T-cell receptor signaling in ALCL tumors (50).

Finally, the WHO-HAEM5 incorporated findings from a recent study by Fitzpatrick et al. that identified *JAK2* fusions in 6% of systemic CD30+ ALK-negative T-cell lymphomas (n=97) (51). Among the cases with JAK2 translocations, three fulfilled the diagnostic criteria of ALK-negative ALCL, and the other three were diagnosed as CD30+ PTCL-NOS. All the cases with *JAK2* translocations featured at least a proportion of ALCL-like large anaplastic tumor cells, in addition to Reed-Sternberg (RS)-like cells. Co-expression of CD30 and CD15 was present in the large anaplastic component in 80% of the cases. In all instances, the tumor cells were negative for PAX-5, and T-cell clonality was established. Due to the limited number of cases, a comparison of differential clinical outcomes was not possible (51). The presence of RS-like cells with co-expression of CD30 and CD15 can be a diagnostic pitfall, and recognition of this subset of cases is critical during diagnosis.

#### 4.1.1 Breast implant-associated ALCL

Although morphologically and immunophenotypically indistinguishable from ALK-negative ALCL, is a distinct clinical entity primarily associated with textured breast implants, generally associated with an indolent clinical course. It is a rare disease with an estimated risk of 1 case in 4000-30,000 women undergoing breast implant surgeries. The median interval from surgery to the development of BIA-ALCL is 8-11 years (52). Diagnosis is often made in the peri-implant effusion fluid as the initial specimen. Cytospin preparations from the fluid show large, pleomorphic tumor cells with prominent nucleoli, abundant vacuolated cytoplasm, and irregular cytoplasmic membranes. Hallmark cells seen in other ALCL types are frequently present. Capsulectomy specimens show varying degrees of infiltration by the tumor cells. In the early stage, they often line the capsule, whereas, in the most advanced stage, they infiltrate through the fibrous capsule to form a mass. Axillary lymph nodes can be involved in approximately 20-30% of cases (53). Capsular invasion, mass formation, and lymph node involvement are adverse prognostic factors (53, 54).

In the updated WHO-HAEM5, BIA-ALCL was upgraded to a definite entity based on its unique clinical, genomic, and molecular features. A comprehensive study of the genetic subtype of BIA-ALCL cases by Feldman et al. (55) shows that there is oncogenic activation of the *JAK-STAT3* signaling pathway caused by mutations of somatic mutations of *STAT3, JAK1*, and *JAK2*. Laurent et al. (56) also demonstrated that the genomic alterations in BIA-ALCL include loss-of-function modifications of the epigenetic modifiers *KMT2C, KMT2D, CHD2*, and *CREBBP*, in up to 74% of cases.

The WHO-HAEM5 highlighted the role of allergic inflammation mediated by the secretion of IL-13 in the pathogenesis of BIA-ALCL (57). The frequent presence of eosinophils and mast cells in the tumor microenvironment of BIA-ALCL further supports the role of allergic inflammation during the pathogenesis of the disease (58, 59).

Finally, WHO-HAEM5 incorporated the role of immune evasion during the disease progression of BIA-ALCL. Tabanelli et al. (60) evaluated PD-L1 and pSTAT expression and PD-L1 copy number alterations (CNAs) in a cohort of 9 BIA-ALCL cases. The findings indicated that 56% of BIA-ALCL overexpressed PD-L1, and 33% of cases harbored CNAs at 9p24.1. Consistent with this, the results also demonstrated variable proportions of PD1+ T-cells and PD-L1+ tumor-associated macrophages (TAMs) in both PD-L1+ and PD-L1-negative BIA-ALCLs, suggesting the presence of an active PD-1/PD-L1 axis (60).

### 4.2 Nodal T-follicular helper type PTCLs

Peripheral T-cell lymphomas derived from T-follicular helper (T-FH) lymphocytes encompass a spectrum of aggressive neoplasms with poor clinical outcomes (61). Two entities in this group are characterized by specific morphological features: angioimmunoblastic T-cell lymphoma (AITL) and Follicular T-cell lymphoma (F-TH). A third group comprises neoplastic expansions of T-follicular helper T-cell lymphocytes that do not fit the characteristic morphological patterns of AITL and T-FH lymphoma. This group was defined in the latest WHO-HAEMR4 as Nodal Peripheral T-cell lymphoma with TFH phenotype (PTCL-TFH).

**Figure 5:**
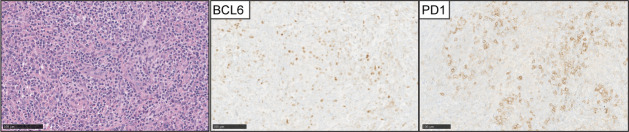
Angioimmunoblastic T-cell lymphoma (AITL). (Left panel) H&E images show characteristic branching of post-endothelial venules and adjacent lymphocytes with clear cell cytoplasm. Expression of BCL-6 and PD1 is detected in the neoplastic T-cell lymphocytes in the majority of cases.

AITL is the second most common type of nodal T-cell lymphoma and accounts for 15-20% of peripheral T-cell lymphomas. Morphologically is characterized by effacement of the nodal architecture, with an expansion of arborizing post-endothelial venules with clusters of neoplastic lymphocytes with clear cytoplasm (**Figure 5**). These areas characteristically feature an expansion of follicular dendritic cell meshworks. The diagnosis can be difficult because the tumor cells represent a minority of the infiltrates, and the tumor microenvironment features positive RS-like cells for EBV with eosinophilic infiltrates (62).

#### 4.2.1 Follicular T-cell lymphoma

Is morphologically distinct from AITL as it doesn’t feature expanded post-endothelial venules surrounded by T-cell lymphocytes with clear cytoplasm. In contrast, two main architectural patterns are described; the most common pattern resembles progressive transformation of the germinal centers (PTGC-L), where the atypical lymphocytes are expanded in poorly defined nodules that are surrounded by numerous B-cell lymphocytes. The second pattern is characterized by well-demarcated nodular expansion of atypical lymphocytes, resembling B-cell follicular lymphoma. Consistent with a T-FH immunophenotype, the atypical cells are CD4 positive and feature co-expression of ICOS, PD1, CD10, BCL-6, and CXCL13; importantly, the expression of one or two of the T-FH markers may be absent. CD21 and CD23 highlight distorted follicular dendritic cell (FDC) meshwork’s overlapping the atypical cells; in contrast to AITL, the FDCs are not expanded or associated with proliferating post-endothelial cells (63–66).

#### 4.2.2 Nodal Peripheral T-cell lymphoma with follicular helper phenotype

**Figure 6:**
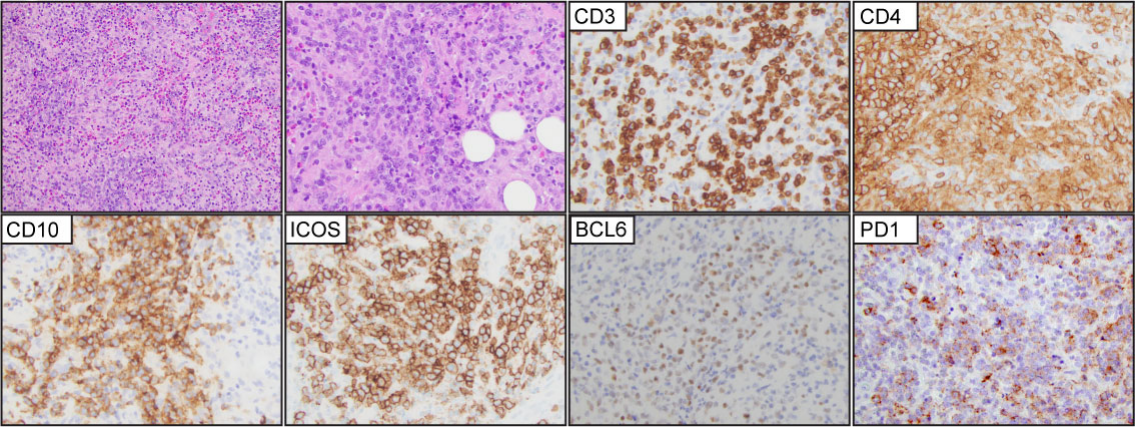
T-follicular helper type peripheral T-cell lymphoma (PTCL-TFH). Representative (H&E) images demonstrate effacement of the nodal architecture by medium to large, atypical lymphocytes that are organized in small clusters with numerous background histiocytes and eosinophils. The tumor cells are positive for CD3, CD4, CD10, ICOS, BCL6, and PD1.

Was a new disease entity introduced in WHO-HAEM4R. This group of cases does not feature the characteristic morphological features of AITL or F-TH lymphoma (**Figure 6**). However, the expression of at least two (preferentially 3) T-FH markers in addition to CD4 is required for classification. ICOS and PD-1 are expressed in most cases. However, ICOS is expressed in 43-52% of the PTCL-NOS cases, and PD-1 is expressed in approximately 60% of PTCL-NOS cases (67, 68). CD10, BCL6, and CXCL13 are the most specific markers and rarely are expressed in PTCL-NOS cases (68).

The family of peripheral T-cell lymphomas with a T-follicular helper (T-FH) origin share similar clinical features and are characterized by disseminated disease at diagnosis with a 5-year overall survival of 30-35% (61). The morphological features can transition between T-FH subtypes over subsequent biopsies or transform into PTCL-NOS. The genetic profile includes similar frequencies of mutations in *DNMT3A*, *RHOA*, and *TET2 (* 61, 69, 70), and *IDH2* mutations are predominantly detected in AITL. The gene expression profiles are similar, and these groups of neoplasms cluster in proximity and apart from other peripheral T-cell lymphomas, including ALCL and PTCL-NOS (69). However, recent studies show that a subset of PTCL-TFH cases features gene expression profiles closer to the PTCL-NOS group (69). To emphasize that this group of neoplasms constitutes a spectrum with morphological plasticity, this family of neoplasms has been designated nodal T-follicular helper lymphomas (nTFH) in the updated WHO-HAEM5. Therefore, the previous designations of ‘angioimmunoblastic T-cell lymphoma,’ ‘follicular T-cell lymphoma,’ and ‘nodal peripheral T-cell lymphoma with TFH phenotype’ will be renamed nTFHL angioimmunoblastic type (nTFHL-AI), nTFHL follicular-type (nTFHL-F) and nTFHL non-otherwise specified (nTFHL-NOS).

### 4.3 Peripheral T-cell lymphoma, non-otherwise specified

This group remains the most prevalent in the United States and constitutes a ‘waste-basket’ category when other disease entities are excluded from the current classification schemes. Recent study series that correlated clinicopathological features with genomic/molecular data has helped to identify distinct groups that are no longer included within this classification, such as Nodal EBV+ T/NK-cell lymphomas and Nodal T-follicular helper lymphomas, NOS.

**Figure 7:**
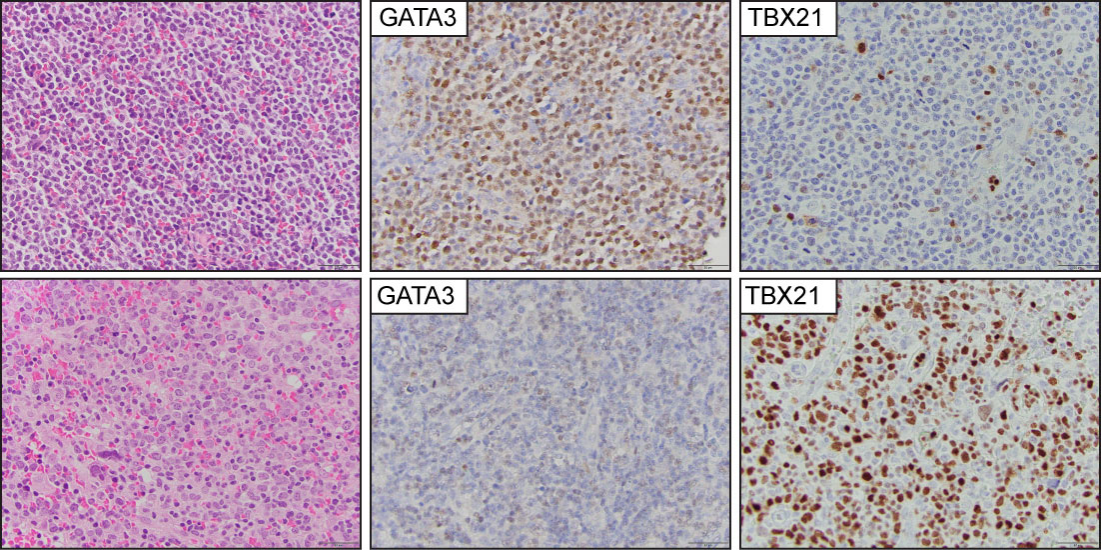
Peripheral T-cell lymphoma, non-otherwise specified (PTCL-NOS). Representative images from two PTCL-NOS cases. The upper panel shows a case classified in the GATA3 positive group, and (the lower panel) highlights a case within the TBX21 group.

Previous studies demonstrated that the expression of the transcription factor GATA-3 is enriched in a subset of PTCL-NOS cases with characteristic transcriptional profiles, worse overall survival, and resistance to chemotherapy (71–74). A second, albeit more heterogeneous subtype, highly expresses the transcription factor TBX21 and is similarly enriched for its gene targets. An immunohistochemistry algorithm that includes CD183, CCR4, GATA-3, and TBX21 has been proposed to identify these cases (75) (**Figure 7**). The upregulated expression of GATA-3 is secondary to the engagement of T-cell receptors in the neoplastic cells, and this is likely secondary to specific interactions with the tumor microenvironment (76–78). Due to insufficient clinicopathological and prognostic findings, the current WHO-HAEM5 did not recognize this group of tumors as a specific subtype of PTCL.

## 5 Epstein-Barr virus positive T-cell lymphomas

The contribution of EBV during the development of lymphoproliferative diseases is well-recognized (79). Importantly, EBV-infected B-cell or T-cells can be identified in the tumor microenvironment of several T-cell lymphomas, including AITL, ATLL, or PTCL-NOS. However, those are likely the result of dysregulated immunosurveillance derived from the primary lymphoma rather than a primary EBV-driven mechanism.

In the setting of immunosuppression, EBV+ T-cell lymphomas are not as common as their B-cell counterparts. However, T-cell lymphoproliferative disorders that occur secondary to EBV infection in immunocompetent hosts are characterized by markedly aggressive behavior and many of those feature dismal outcomes. This category includes T and NK-cell-derived lymphoproliferative disorders in the setting of chronic active EBV infection (T/NK type CAEBV). T/NK type CAEBV will feature EBV+ T or NK-cell expansions that do not feature definitive morphological or immunophenotypic features compatible with a T or NK-cell type lymphoma diagnosis. However, the clinical course is very similar to T-cell lymphomas and is characterized by hepatitis, vasculitis, hemophagocytic syndrome, and end-organ damage (80–84). Aggressive NK-cell leukemia (ANKL) also features EBV+ lymphoma cells and is discussed in a different section (2.5). The WHO-HAEM5 has incorporated novel molecular findings in extranodal T/NK type lymphoma (ENKTL) and included nodal EBV+ T/NK-cell lymphoma as a new disease entity.

### 5.1 Extranodal NK/T-cell lymphoma, nasal type

**Figure 8:**
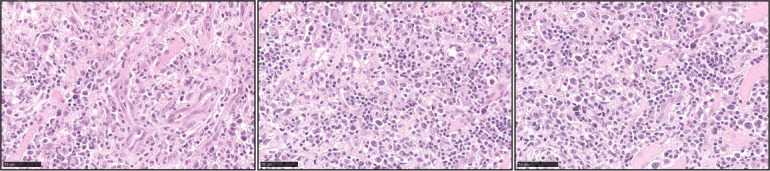
Extranodal T/NK-cell lymphoma, nasal type (ENKTL). Three representative pictures of ENTKL (H&E) highlight the pleomorphism of the atypical infiltrates. The atypical infiltrates can be predominantly composed of small lymphocytes or medium to large atypical forms. Angiocentricity is commonly identified.

ENKTL is an aggressive type of lymphoproliferative disorder predominantly derived from NK cells, with a minority of cases from cytotoxic T-cell origin. Approximately 80% of the cases are localized in the nasal cavity or nasopharynx (85). The remaining 20% of the cases will involve other sites that include skin, testicles, and salivary glands. Morphologically the neoplastic infiltrate can show a broad spectrum of appearances ranging from small cells with minimal atypia to medium to large pleomorphic cells with marked cellular atypia (86, 87) (**Figure 8**). Necrosis is observed in approximately 60%-80% of the cases, and angiocentricity is present in nearly 50% of the cases (85, 86, 88). Virtually all the cases are EBV+ in more than 80% of the tumor cells (85, 87, 88). An NK-cell immunophenotype is the most frequently observed and is characterized by negative expression of surface CD3, with positive expression of cytoplasmic CD3ε, CD56, and the cytotoxic markers TIA-1 and granzyme B. Approximately 20% of true NK-cell origin tumors (germline TCRγ) will feature negative expression of CD56, however, expression of cytotoxic markers is always present (87–89). Cases with a T-cell origin defined by monoclonal TCRγ rearrangement can account for 20%-40%, and approximately 40% of those will lack CD56 expression (85, 89). However, EBV negative cases with negative expression of CD56 should be designated as PTCL-NOS (87). In recognition that a subset of these cases involves extra-nodal sites other than the nasal cavity, the WHO-HAEM5 has removed the term ‘nasal-type’ from this designation. In addition, the WHO-HAEM5 mentioned novel genomic findings that highlight the relevance of *STAT3* mutations in ENKTL and the therapeutic role of PD-L1.

A recent study by Song et al. conducted targeted gene capture sequencing in 171 PTCLs, and *JAK*/*STAT* mutations were identified in 78% of the ENKTL cases (n=109) (90). Mutations in *STAT3* were predominantly detected in ENKTL cases (21%), in contrast to ALCL (3.7%) and PTCL-NOS cases (3.8%). The mutations identified in *STAT3* were associated with increased transcriptional activity and higher expression of PD-L1 in-vitro. However, the evaluation of primary ENKTL samples showed that 93% of the cases (n=30) featured increased expression of PD-L1, suggesting that additional molecular mechanisms are involved in PD-L1 expression (90). Consistent with this, Bi et al. demonstrated that the expression of the EBV latent membrane protein 1 (LMP1) promotes PD-L1 expression in ENKTL cell lines in-vitro (91).

Finally, the therapeutic potential of PD-L1 in ENKTL has been evaluated in a recent phase 2 clinical trial that tested the efficacy of the IgG1-PD-L1 antibody avelumab as a single agent in refractory/relapsed (R/R) ENKTL (92). This study by Jin et al. demonstrated a 24% complete response rate in R/R patients that received avelumab (92). The patient responses to avelumab were associated with higher expression of PD-L1 in the tumor cells. However, response rates were not related to the levels of serum soluble PD-L1 (92).

### 5.2 Nodal EBV-positive T and NK-lymphomas

This novel designation encompasses EBV+ NK/T cell lymphomas with a predominant nodal presentation. These tumors are characteristically diagnosed at an advanced stage and feature dismal clinical outcomes with overall survival of 1.5-3.5 months after diagnosis (93–95). This new entity differs from ENKTCL with secondary nodal involvement, as the former will primarily be present at extranodal sites. Cases of chronic active EBV infection of T-cell or NK-cell type (T/NK- type CAEBV) are also excluded from this category, as those do not feature morphological features consistent with a lymphoproliferative neoplasm. Finally, the presence of leukemic involvement by neoplastic cells with an NK-cell immunophenotype will be best classified as ANKL.

Other nodal T-cell lymphomas can feature a subset of tumor cells positive for EBV. Therefore, the minimal threshold for the number of EBV+ neoplastic cells varies among the published case series and ranges between 40%-50% (93–95). Immunophenotypically, approximately 60% of the cases will show tumor cells with a T-cell immunophenotype that are positive for CD8 and TβF1. The tumor cells will be less frequently positive for CD4 (93–95). Tumor cells with a characteristic NK-cell immunophenotype have been described. However, those account for less than 10% of the cases described (93, 94, 96).

This group of neoplasms features gene-expression profiles distinct from ENKTL with frequent loss of 14q11.2 and upregulation of PD-L1 (CD174) (93). Functional analysis of the gene-expression profiles demonstrates enrichment for NF-κB signaling (97). Consistent with this and in contrast to PTCL-NOS cases, predominant expression of BIRC3 and p50 is detected (97). Frequent mutations in *TET2* (64%, n=14), followed by *PIK3CD* (33%, n=14) and *STAT3* (19%, n=14) (97) characterize the genomic landscape of EBV+ NTNKCL.

## 6 Hepatosplenic T-cell lymphoma

Hepatosplenic T-cell lymphoma (HSTCL) is a rare but aggressive type of extranodal T-cell lymphoma that accounts for 1-2% of all peripheral T-cell lymphomas. The tumor cells involve the liver, spleen, and bone marrow in an exclusive intrasinusoidal distribution, often engorging and distending the cords and sinuses in the involved organs (39, 98). The morphology of the atypical infiltrates is characterized by monotonous, medium-sized lymphocytes with irregular nuclear contours, a moderate amount of agranular pale cytoplasm, and inconspicuous nucleoli. In a subset of the cases, the neoplastic cells feature blastoid chromatin, especially at advanced stages (99–101). Immunophenotypically, the neoplastic cells express pan T-cell markers CD2, CD3, and CD7, with frequent co-expression of CD56 and aberrant loss of CD5. A subset of cases is CD8+. However, the majority are negative for both CD4 and CD8. Expression of the cytotoxic granule-associated proteins, TIA1, and granzyme M is commonly observed. Expression of TCR-γ/δ is frequently observed (~75%), while ~25% of cases are TCR-α/β, and in a small number of instances (~5%), TCR-silent (102).

Several cytogenetic abnormalities are reported in HSTCL. The most common are isochromosome 7q [i(7q)] and trisomy 8 and are found in approximately 63% and 50% of cases, respectively (103). A unique molecular signature is identified in HSTCL cases. This is characterized by overexpression of genes encoding NK-cell-associated molecules (*FOS* and *VAV3*), the sphingosine-1-phosphatase receptor 5 (*S1PR5*) involved in cell trafficking, the tyrosine kinase *SYK*, with down-regulation of tumor suppressor gene *AIM1* (104).

While HSTCL predominantly occurs in immunocompetent individuals, 20-30% of cases occur in chronic immune suppression or immune dysregulation associated with autoimmune disorders (99, 100, 105). The WHO-HAEM4 reported that this disease is mainly diagnosed in adolescents and young adults. However, a recent report from the prospective T-cell lymphoma project identified that more than 50% of the patients in their cohort were older than 60 years old. Thus, the WHO-HAEM5 acknowledged that the disease is diagnosed in adolescents, young adults, and older individuals (98).

The WHO-HAEM5 also incorporated findings from a recent study by Mckinney et al. (106) that analyzed the genomic landscape of a large cohort of HSTCL cases with whole-exome sequencing. The results demonstrated frequent mutations in genes involving the JAK/STAT signaling pathway, with *STAT3* and *STAT5B* being the most frequently mutated. In addition, mutations in *PI3KCD* and the epigenetic regulators *SETD2*, *INO80*, *TET3*, and *SMARCA2* were observed in up to 62% of the cases (52, 106, 107).

## 7 Conclusions

The diagnosis and classification of T-cell lymphomas require a correlation between the histomorphology, molecular studies, and the site and manner of presentation. To date, few entities such as ALK+ ALCL or T-PLL display specific genetic aberrancies that are the predominant driving force of lymphoma progression and therefore constitute a distinctive feature for classification. In contrast, most T-cell lymphomas share morphological similarities and feature different frequencies of genetic alterations that either amplify the T-cell receptor signaling (e.g., NF-kB, RhoA, GATA3) or bypass it entirely (e.g., JAK/STAT, Pi3K). Therefore, a more significant proportion of mature T-cell lymphomas remain diagnosed in the unclassifiable (NOS) group. Currently, there is a limited understanding of the biology of T-cell neoplasms, which translates into limited successful therapeutic approaches, and these tumors remain an area of unmet need.

## Author contributions

KI and CMZ wrote the manuscript. All the authors contributed to the article and approved the submitted version.
